# Real-World Healthcare Resource Utilization and Cost Burden Assessment for Adults With Generalized Myasthenia Gravis in the United States

**DOI:** 10.3389/fneur.2021.809999

**Published:** 2022-01-18

**Authors:** Glenn Phillips, Catarina Abreu, Amit Goyal, Yuebing Li, Albert Whangbo, Deborah Gelinas, Edward Brauer, Sankha Bhattacharya

**Affiliations:** ^1^argenx US Inc., Boston, MA, United States; ^2^ZS Associates, New York, NY, United States; ^3^ZS Associates, Princeton, NJ, United States; ^4^Neuromuscular Center, Cleveland Clinic, Cleveland, OH, United States; ^5^ZS Associates, Bangalore, India

**Keywords:** myasthenia gravis (MG), myasthenic crisis, healthcare resource utilization (HCRU), cost analysis, disease burden

## Abstract

**Introduction:**

Limited evidence exists for healthcare resource utilization (HCRU) and costs associated with generalized myasthenia gravis (gMG), a rare autoimmune disorder, for adults in the United States.

**Methods:**

Adults with ≥1 diagnostic claim for MG between 2014 and 2019 were identified using Symphony Health's Integrated Dataverse®. Using a novel algorithm, HCRU and costs over 12 months following index dates were evaluated for patients with gMG including those with exacerbation events. For patients who experienced crisis events, HCRU and costs were analyzed during the 36 months preceding, during, and 12 months following the events.

**Results:**

Mean HCRU and costs were higher for newly diagnosed patients compared with previously diagnosed patients (hospitalizations: 0.46 vs. 0.34; all-cause costs: $26,419.20 vs. $24,941.47; direct costs for gMG treatments: $9,890.37 vs. $9,186.47) and further increased for patients with exacerbation events (hospitalizations: 0.72; all-cause costs: $43,734.15; direct costs for gMG treatments: $21,550.02). For patients who experienced crisis events, HCRU and costs markedly increased during the 12 months immediately before the crisis event (hospitalizations: 1.35; all-cause costs: $49,236.68) compared with the 2 preceding years and increased further during the 12 months following the crisis index date (hospitalizations: 2.78; all-cause costs: $173,956.99). Cost increases were, in large part, attributed to treatments received.

**Discussion:**

New diagnosis, exacerbation, and crisis events were drivers of HCRU and cost for patients with gMG. Particularly, high costs of gMG-specific medications associated with intervention for exacerbation and crisis events contributed to increased all-cause costs.

## Introduction

Myasthenia gravis (MG) is a rare autoimmune disorder associated with the failure of neuromuscular junction (NMJ) transmission and is characterized by autoantibodies that target specific proteins involved in NMJ signaling (1). The most common autoantigen is the acetylcholine receptor; other autoantigens involved in NMJ formation and maintenance (e.g., MuSK [muscle-specific kinase] and LRP4 [low-density lipoprotein receptor-related protein 4]) have been identified in a subset of patients (1). In the United States (US), prevalence is estimated at 14–20 per 100,000 population with ~60,000 patients currently living with MG, though figures are likely higher since MG is often underdiagnosed (2–4). While MG presents with ocular symptoms in approximately two-thirds of patients, symptoms remain isolated to the ocular area in only 15% of cases (referred to as ocular MG or oMG) (1). For the remaining majority of patients, the disease progresses to generalized MG (gMG), which may involve bulbar, limb, trunk, and respiratory muscles (5). Exacerbation of gMG symptoms can lead to a myasthenic crisis defined as respiratory failure requiring mechanical ventilation, which occurs at least once in 15% to 20% of patients with MG during their lifetime (6).

A systematic literature review of the economic burden of MG in the US showed that healthcare resource utilization (HCRU) associated with MG was particularly increased in patients with treatment-refractory MG (defined as receiving multiple or a complex regimen of MG therapies) and patients experiencing crisis events (7–9). However, limited US-based evidence was available for cost burden (10), and available studies were outdated for providing reliable cost estimates—particularly, the cost burden of gMG has not been clearly delineated from that of oMG (11). With the objective of filling essential knowledge gaps, we evaluated HCRU and costs associated with gMG (including myasthenic crisis management) by designing a novel analysis method validated by gMG experts based on data extracted from a comprehensive US claims database. As a secondary objective, we evaluated treatment patterns associated with subgroups of patients with gMG.

## Materials and Methods

### Expert Interviews

Five US-based neuromuscular specialists experienced in gMG management were interviewed individually during 1-h sessions on disease epidemiology, treatment patterns, and clinical practice of gMG. All experts reviewed the initial approaches for the cost analysis, provided input on the methodology, and were engaged throughout the analysis for relevant feedback. Further details on the interview structure can be found in the Supplementary Methods.

### Study Design and Data Source

This retrospective observational study was conducted using Symphony Health's (Blue Bell, PA, USA) Integrated Dataverse (IDV®) of pharmaceutical and medical claims. The IDV® claims database links healthcare data of ~280 million enrollees in the US from pharmacy point-of-service sales, within-network transactions, and additional direct prescriptions (including medical and hospital claims data feeds). The robust database captures a high proportion of prescription transactions across the US and includes information from a range of payment types including commercial insurance, Medicare, Medicaid, and cash. At the authors' request, Symphony Health (Blue Bell, PA, USA) identified study patients spanning the period of January 1, 2014 to December 31, 2019, and relevant data were licensed by authors through ZS Associates' (Evanston, IL, USA) data partnership agreement with Symphony Health (Blue Bell, PA, USA). The de-identified claims data included details on patient prescriptions and diagnoses as well as procedural, surgical, and health service data, and the settings in which they were administered. NDCs (National Drug Codes) and CPT® (Current Procedural Terminology®) codes were used to identify therapies, services, and procedures received by patients across outpatient, inpatient, and other care settings from the extracted dataset. No identifiable or protected health information was obtained for use in this study.

### Patient Selection

#### Inclusion Criteria

Adults (≥18 years of age) with claims including International Classification of Diseases, Ninth or Tenth Editions (ICD-9 or ICD-10) diagnostic codes associated with MG were included in the initial screening (Supplementary Table 1). Patients with ≥2 such claims filed at least 1 month apart between January 1, 2014 and December 31, 2019 were selected to limit cases involving misdiagnoses. Further, patients with MG diagnostic claims filed only by ophthalmologic specialists (defined as ophthalmologists, pediatric ophthalmologists, or optometrists) were considered more likely to be diagnosed with oMG instead of gMG and were excluded from the analysis. The final study cohort included patients who had at least 1 claim filed between January 1, 2017 and December 31, 2018 (Supplementary Figure 1).

#### Subgroup Definitions

Detailed inclusion and exclusion criteria for each subgroup are summarized in Supplementary Table 2. Of the final study cohort, patients who were newly diagnosed during the study period were distinguished from patients who received their first diagnosis prior to the study period according to claims data. Patients who fulfilled the criteria for the exacerbation and crisis event subgroups were drawn from the final study cohort regardless of the time of their first diagnosis. The crisis event subgroup was defined by the presence of one or more intubation claims with an associated inpatient stay and ICU (intensive care unit) admission. The exacerbation event subgroup was defined only by the presence of MG exacerbation ICD codes G70.01 (ICD-10) or 358.01 (ICD-9) with a concomitant absence of intubation claims. Of note, reasons for the exacerbation claim and the accuracy of coding practices was indiscernible in the claims dataset (limitations are further addressed in the Discussion).

### Time Period Selection

Each patient was associated with a gMG index date defined as the first occurrence of a diagnostic claim for MG filed by a non-ophthalmologic specialist between January 1, 2017 and December 31, 2018. Patients were considered newly diagnosed (ND) if the index date was their first diagnosis of MG in the available data, and previously diagnosed (PD) if their first diagnosis of MG in the available data occurred before the index date; the analysis period continued for 12 months following the index dates.

For the exacerbation event subgroup, the exacerbation index date was defined as the date of first acute exacerbation claim filed between January 1, 2017 and December 31, 2018; the analysis period continued for 12 months following the exacerbation index date.

For the crisis event subgroup, the crisis index date was defined as the date of first intubation claim filed between January 1, 2017 and December 31, 2018. The analysis period for the crisis event subgroup spanned across three time periods: pre-crisis, during the crisis, and post-crisis. The pre-crisis time period was defined as up to 3 years preceding the crisis index date (with intervals of 1 year). The crisis event start date was defined as the date of intubation (crisis index date), and the end date was defined as the last date of a continuous inpatient stay. The post-crisis time period was defined as 12 months following the crisis index date; thus, post-crisis time periods include the crisis event duration.

For each time period, continuous quarterly activity (defined as ≥1 claim filed per quarter) was assessed for missing data; patients were included in the cost analysis if they had at least 1 claim activity of any type (MG or non-MG) during each quarter within the time period of interest. December 31, 2018 was chosen as the end date for patient selection to allow a follow-up period of 12 months from diagnosis that ended before the beginning of the COVID-19 (coronavirus disease 2019) pandemic.

### Study Measures

#### Baseline Demographics

Baseline demographics at the index date were analyzed for the final study cohort and subgroups. Parameters notated in the claims data included age, gender, comorbidities, and health insurance plan type at the index date. Mean ± standard deviation (SD) and median age were derived from patients' year of birth. Health insurance and plan type were defined as commercial, Medicare, Medicaid, or other. The five most frequently occurring comorbidities per ICD-10 diagnostic codes (excluding gMG-related diagnostic codes) within patients' claims were assessed.

#### Healthcare Resource Utilization

HCRU for medical services was evaluated using the place of service and procedure codes listed for each claim in the dataset. Visits were broadly classified into hospitalizations, outpatient services (defined as hospital outpatient and clinic visits), emergency department (ED) visits, office visits, and other services. Hospitalizations were defined as the continuous stay of a patient in a hospital/inpatient setting (identified on basis of place of service in the data), and mean hospitalizations per patient were calculated using distinct instances per patient for continuous inpatient stays. Outpatient, office, and other visits were determined using the unique number of corresponding claims and calculating mean visits per patient for each category. Mean ED visits per patient were identified using claims with procedure code descriptions for ED, critical care, hospital observation, and emergency service settings.

#### Treatment Pattern Analysis

Claims including treatment with intravenous immunoglobulin (IVIg) or subcutaneous immunoglobulin (SCIg), rituximab, eculizumab, plasma exchange (PLEX), acetylcholinesterase (AChE) inhibitors, non-steroidal immunosuppressive treatments (NSISTs), or corticosteroids were considered gMG-related treatments and included in treatment pattern analyses. NSISTs included azathioprine, cyclophosphamide, cyclosporine, methotrexate, mycophenolate, and tacrolimus.

#### Cost Analysis

Estimated paid amounts were derived from charged amounts for medical procedures provided in the IDV® dataset. Mean and total costs were evaluated for a 12-month period at the patient level from the payer perspective, and subset analyses were performed by separating total costs into medical service costs and pharmacy costs as detailed in the Supplementary Methods.

Direct costs for gMG treatments were estimated based on costs incurred for the following gMG-relevant therapies: standard-of-care therapies (AChE inhibitors, NSISTs, and corticosteroids) and add-on therapies (IVIg or SCIg, rituximab, eculizumab, or PLEX).

For the ND, PD, and exacerbation event subgroups, mean, total, medical, and pharmacy costs were estimated across the outpatient, inpatient, clinic, office, and other/unknown settings 12 months after the index date. For the crisis event subgroup, mean, total, medical, and pharmacy costs were analyzed annually for up to 3 years before the crisis event index date, during the crisis event, and 12 months following the crisis index date. All costs were standardized to 2018 US dollars using the CPI-U (Consumer Price Index for All Urban Consumers) provided by the US Bureau of Labor Statistics (https://data.bls.gov/timeseries/CUUR0000SAM?output_view=data). Further details on cost estimation methods are available in the Supplementary Methods.

### Statistical Analysis

For baseline patient demographic and clinical characteristics in the overall cohort and patient subgroups, continuous variables were summarized using mean and SD, while binary and categorical variables, such as gender, insurance type, comorbidities, patients with ≥1 filed claim, and treatment patterns were described using frequencies and percentages. HCRU and costs were calculated as standardized mean (total HCRU or costs divided by number of patients in the cohort).

For comparisons, two sample *t*-tests were used to assess statistically significant differences in mean age between male and female patients. HCRU related to percentage of patients with ≥1 filed claim and treatment patterns in ND and PD patient subgroups were compared using Chi-squared tests. As data were not normally distributed, HCRU related to hospital visits and length of stay (LOS) in ND and PD subgroups were compared using Wilcoxon rank sum tests. For comparisons between time periods within the crisis event subgroup, McNemar tests and Wilcoxon signed-rank tests were used to account for dependency of data within the same individuals.

Analyses were conducted using R 4.0.4 (R Foundation for Statistical Computing, Auckland, New Zealand), and statistical significance was assessed at the 0.05 level. Detailed statistical analysis results are reported in the Supplementary Tables 2, 3, 5, 8, and 9.

## Results

### Study Cohort

Of patients with claims including MG diagnostic codes between January 1, 2014 and December 31, 2019 identified in the dataset, 66,119 patients who fulfilled the study inclusion criteria and had at least 1 claim filed between January 1, 2017 and December 31, 2018 were further screened. From this cohort, 1,560 patients whose claims were associated with only ophthalmologic specialists were excluded from further analysis. Of the 41,490 total patients with continuous quarterly claims activity included in the final study cohort, 12,822 patients were identified as ND and 29,118 patients were identified as PD with gMG prior to the study period. Regardless of the time of first diagnosis, 4,355 patients fulfilled criteria for the exacerbation event subgroup and 206 patients for the crisis event subgroup (Supplementary Figure 1).

### Baseline Demographics

Table 1 summarizes baseline demographics of the final study cohort and subgroups at the index date. Mean (SD) age of the total study cohort was 64.78 (13.43) years (median [interquartile range]: 69 [58–77] years). Mean age of female patients (61.75 years) was significantly lower than that of male patients (68.07 years; *p* < 0.001), suggesting female patients were diagnosed at a younger age.

**Table 1:** Baseline patient demographic and clinical characteristics at index date.

**Baseline characteristics**	**Total (*N* = 41,940)**	**ND (*n* = 12,822)**	**PD (*n* = 29,118)**	**Exacerbation event (*n* = 4,355)**	**Crisis event (*n* = 206)**
**Age, years**					
Mean (SD)	64.78 (13.43)	65.46 (12.89)	64.48 (13.66)	65.57 (12.89)	63.70 (14.89)
Median (IQR)	69 (58–77)	69 (59–77)	69 (57–77)	69 (59–77)	68 (56–76)
Gender, n (%)^*^					
Male	20,116 (47.96)	6,325 (49.33)	13,791 (47.37)	2,047 (47.00)	95 (46.12)
Female	21,823 (52.04)	6,496 (51.66)	15,327 (52.63)	2,308 (53.00)	111 (53.88)
Unknown	1 (0)	1 (0.01)	0 (0)	0 (0)	0 (0)
Mean age by gender, years (SD)^*^					
Male	68.07 (10.82)	68.20 (10.37)	68.00 (11.02)	68.62 (10.07)	68.58 (9.71)
Female	61.75 (14.83)	62.79 (14.45)	61.31 (14.97)	62.87 (14.43)	59.52 (17.13)
**Health insurance type, n (%)** ^**†**^					
Commercial	33,912 (80.86)	10,419 (81.26)	23,493 (80.68)	3,641 (83.60)	188 (91.26)
Medicare	23,975 (57.16)	7,365 (57.44)	16,610 (57.04)	2,645 (60.70)	133 (64.56)
Medicaid	4,361 (10.40)	1,240 (9.67)	3,121 (10.72)	489 (11.20)	39 (18.93)
Other^‡^	3,797 (9.05)	1,208 (9.42)	2,589 (8.89)	456 (10.50)	31 (15.05)
**Pharmacy insurance type, n (%)** ^**†**^					
Commercial	18,823 (44.88)	5,791 (45.16)	13,032 (44.76)	1,953 (44.80)	90 (43.69)
Medicare	20,282 (48.36)	6,301 (49.14)	13,981 (48.01)	2,230 (51.20)	107 (51.94)
Medicaid	5,103 (12.17)	1,527 (11.91)	3,576 (12.28)	570 (13.10)	36 (17.48)
Other^‡^	12,909 (30.78)	4,263 (33.25)	8,646 (29.69)	1,563 (35.90)	82 (39.81)
**Top five comorbidities (ICD-10 code), n (%)** ^**§**^					
Essential hypertension (I10)	22,928 (54.67)	7,242 (56.48)	15,686 (53.87)	2,590 (59.47)	173 (83.98)
Hyperlipidemia, unspecified (E78.5)	12,415 (29.60)	4,006 (31.24)	8,409 (28.88)	1,416 (32.51)	91 (44.17)
Type 2 DM without complications (E11.9)	10,287 (24.53)	3,176 (24.77)	7,111 (24.42)	1,275 (29.28)	97 (47.09)
GERD without esophagitis (K21.9)	8,829 (21.05)	2,828 (22.06)	6,001 (20.61)	1,041 (23.90)	79 (38.35)
Hypothyroidism, unspecified (E03.9)	7,829 (18.67)	2,482 (19.36)	5,347 (18.36)	933 (21.42)	42 (20.39)

*DM, diabetes mellitus; GERD, gastroesophageal reflux disease; ICD, International Classification of Diseases; IQR, interquartile range; ND, newly diagnosed; PD, previously diagnosed; SD, standard deviation*.

* *Claims data denote gender, as opposed to sex*.

† *Percentages may exceed 100% as some patients were covered under more than one plan type*.

‡ *Other includes cash, government, pharmacy benefit managers, and unknown*.

§ *Percentages may exceed 100% due to a subset of patients having multiple comorbidities*.

Commercial plans and Medicare were the most common health insurance types observed among the total study cohort, while pharmacy insurance was spread between commercial plans, Medicare, and other sources. Medicaid was less utilized among the study cohort across both health and pharmacy insurance plans compared with Medicare and commercial plans.

The most frequent comorbidity identified was essential hypertension followed by hyperlipidemia, type 2 diabetes mellitus, gastroesophageal reflux disease (GERD), and hypothyroidism (Table 1; Supplementary Table 3). While the prevalence of hypertension (12), diabetes (13), and GERD (14) within the study cohort were largely consistent with age-matched national reporting, the occurrence of hyperlipidemia (15) and hypothyroidism (16) trended higher in the study cohort compared with data based on a general population. Notably, all 5 of the most frequent comorbidities were overrepresented among patients in the crisis event subgroup compared with the total study cohort.

### Healthcare Resource Utilization

Compared with PD patients, ND patients had significantly higher HCRU with greater mean hospitalizations, longer mean LOS, and increased mean ED, outpatient, and office visits over 12 months (*p* < 0.001) (Table 2). A large proportion of ND patients with hospitalizations was observed in the first quarter of the year following the index date, which then stabilized over the remaining quarters (Supplementary Table 4).

**Table 2:** Standardized healthcare resource utilization in ND, PD, and exacerbation event subgroups over 12 months.

**Healthcare resource unit**	**ND (*n* = 12,822)**	**PD (*n* = 29,118)**	**Exacerbation event (*n* = 4,355)**
**Hospitalizations**			
Patients with ≥1 filed claim, n (%)	3,032 (23.64)	5,472 (18.79)	1,569 (36.02)
12-month hospitalizations, standardized mean	0.46	0.34	0.72
LOS (days), standardized mean	1.39	0.99	2.04
**ED visits**			
Patients with ≥1 filed claim, n (%)	4,023 (31.37)	8,415 (28.90)	1,661 (38.41)
12-month ED visits, standardized mean	1	0.88	1.24
**Outpatient visits**			
Patients with ≥1 filed claim, n (%)	9,465 (73.81)	20,707 (71.11)	3,250 (74.62)
12-month outpatient visits, standardized mean	7.33	6.92	8.76
**Office visits**			
Patients with ≥1 filed claim, n (%)	10,751 (83.84)	24,110 (82.80)	3,576 (82.11)
12-month office visits, standardized mean	8.76	7.98	9.49
Other visits^*^			
Patients with ≥1 filed claim, n (%)	6,224 (48.54)	14,114 (48.47)	2,516 (57.77)
12-month office visits, standardized mean	4.25	4.57	6.59

*ED, emergency department; LOS, length of stay; ND, newly diagnosed; PD, previously diagnosed*.

* *Other visits included care provided in settings that did not fall within other defined categories such as independent laboratories, home health agencies, and hospices*.

HCRU was further increased for patients who experienced exacerbation events, with markedly higher values observed across hospitalizations, LOS, ED, outpatient, and office visits compared with the larger ND or PD cohorts (*p* < 0.001) (Table 2). For patients who experienced crisis events, HCRU during the 36–25 months and 24–13 months leading up to the crisis index date was comparable to or lower than that observed in the larger ND or PD cohorts; however, a dramatic increase in HCRU was observed during the 12 months immediately preceding the crisis event (*p* < 0.001) (Table 3). During this time period, more than a 2-fold increase in mean hospitalizations, LOS, and ED visits was observed compared with the two preceding years. As expected, mean LOS and ED visits markedly increased during crisis events. In the 12-month period following the crisis index date (which included the crisis event duration), mean hospitalizations, LOS, and ED visits increased 2- to 3-fold further when compared with the 12-month period preceding the crisis event (*p* < 0.001).

**Table 3:** Standardized healthcare resource utilization in the crisis event subgroup.

**Healthcare resource unit**	**Pre-crisis (*****n*** **=** **206)**			Crisis event^*^(*n* = 206)	**Post-crisis^**†**^ (*n* = 206)**
	**36–25 months**	**24–13 months**	**12–0 months**		**0–12 months**
**Hospitalizations**					
Patients with ≥1 filed claim, n (%)	47 (22.81)	52 (25.24)	125 (60.68)	206 (100)	206 (100)
12-month hospitalizations, standardized mean	0.46	0.50	1.35	1.00	2.78
LOS (days), standardized mean	1.27	1.47	3.60	15.38	10.14
**ED visits**					
Patients with ≥1 filed claim, n (%)	62 (30.09)	74 (35.92)	133 (64.56)	205 (99.51)	206 (100)
12-month ED visits, standardized mean	1.24	1.34	3.01	5.65	8.78
**Outpatient visits**					
Patients with ≥1 filed claim, n (%)	141 (68.44)	142 (68.93)	169 (82.03)	104^§^ (50.48)	184 (89.32)
12-month outpatient visits, standardized mean	7.00	7.43	11.34	1.26	16.32
**Office visits**					
Patients with ≥1 filed claim, n (%)	156 (75.72)	150 (72.81)	171 (83.00)	12 (5.83)	169 (82.03)
12-month office visits, standardized mean	6.31	6.40	9.31	0.10	10.00
**Other visits** ^**‡**^					
Patients with ≥1 filed claim, n (%)	101 (49.02)	101 (49.02)	133 (64.56)	66 (32.03)	172 (83.49)
12-month office visits, standardized mean	4.02	4.60	6.47	0.66	15.33

*ED, emergency department; LOS, length of stay*.

* *Crisis event HCRU was evaluated during hospital stay*.

† *Post-crisis period included the crisis duration*.

‡ *Other visits included care provided in settings that did not fall within other defined categories such as independent laboratories, home health agencies, and hospices*.

§ *Since visits were calculated based on respective claims, outpatient/office claims present in the data appear proportionately lower during crisis duration*.

### Treatment Pattern Analysis

Treatment patterns between ND and PD patients were similar, with the most frequently used medications being AChE inhibitors, corticosteroids, and NSISTs (Table 4). While patients who experienced exacerbation events used similar standard-of-care therapies, more than double the proportion of patients in the exacerbation event subgroup reported use of rescue or add-on therapies including IVIg or SCIg (17.3%), PLEX (6.5%), and eculizumab (1.2%) compared with the ND and PD cohorts.

**Table 4:** Patient distribution within each drug class for ND, PD, and exacerbation event subgroups.

**Therapeutic class, n (%)**	**ND (*n* = 12,822)**	**PD (*n* = 29,118)**	**Exacerbation event (*n* = 4,355)**
IVIg + SCIg	1,016 (7.9)	2,387 (8.1)	755 (17.3)
Rituximab	99 (0.7)	348 (1.1)	66 (1.5)
Eculizumab	89 (0.6)	58 (0.1)	53 (1.2)
PLEX	354 (2.7)	695 (2.3)	286 (6.5)
AChE inhibitors	6,546 (51.1)	13,394 (45.9)	2,391 (54.9)
NSISTs^*^	3,053 (23.8)	8,222 (28.2)	1,477 (33.9)
Corticosteroids	5,986 (46.6)	12,293 (42.2)	2,344 (53.8)

*AChE, acetylcholinesterase; IVIg, intravenous immunoglobulin; ND, newly diagnosed; NSIST, non-steroidal immunosuppressive treatment; PD, previously diagnosed; PLEX, plasma exchange; SCIg, subcutaneous immunoglobulin*.

* *NSISTs included azathioprine, cyclophosphamide, cyclosporine, methotrexate, mycophenolate, and tacrolimus*.

Treatment patterns during pre-crisis, crisis, and post-crisis periods were analyzed for patients in the crisis event subgroup (Table 5). Consistent with the HCRU increases observed, the proportion of patients receiving AChE inhibitors, corticosteroids, NSISTs, and PLEX significantly increased (*p* < 0.01) in the 12-month period immediately before the crisis event compared with the 2 preceding years, and were maintained or further increased during the 12 months following the crisis index date. Though eculizumab was not used by any patient in this subgroup during the 3 years preceding the crisis event, 2.4% of patients were prescribed eculizumab during the 12 months following the crisis index date. IVIg and SCIg use increased over the 3 years leading to the crisis event (4.4% to 7.8% to 8.7%) with a significant increase observed between the 12 months before and after the crisis index date (14.5%; *p* < 0.05).

**Table 5:** Patient distribution within each drug class for the crisis event subgroup.

**Therapeutic class, n (%)**	**Pre-crisis (*****n*** **=** **206)**			Crisis event^*^(*n* = 206)	**Post-crisis (*n* = 206)**
	**36–25 months**	**24–13 months**	**12–0 months**		**0–12 months**
IVIg + SCIg	9 (4.4)	16 (7.8)	18 (8.7)	2 (1.0)	30 (14.5)
Rituximab	2 (1.0)	4 (1.9)	2 (1.0)	0 (0)	8 (3.9)
Eculizumab	0 (0)	0 (0)	0 (0)	0 (0)	5 (2.4)
PLEX	9 (4.4)	5 (2.4)	13 (6.3)	37 (17.9)	53 (25.7)
AChE inhibitors	67 (32.5)	73 (35.4)	111 (53.8)	19 (9.2)	117 (56.7)
NSISTs^†^	43 (20.9)	39 (18.9)	53 (25.7)	6 (2.9)	82 (39.8)
Corticosteroids	77 (37.4)	88 (42.7)	110 (53.4)	30 (14.6)	120 (58.3)

*AChE, acetylcholinesterase; IVIg, intravenous immunoglobulin; NSIST, non-steroidal immunosuppressive treatment; PLEX, plasma exchange; SCIg, subcutaneous immunoglobulin*.

* *Most costs and drug utilization information during a crisis event were captured from inpatient claims. These inpatient costs are not directly attributable to gMG-specific therapies; hence, overall utilization numbers are underreported (nearly to 45%)*.

† *NSISTs included azathioprine, cyclophosphamide, cyclosporine, methotrexate, mycophenolate, and tacrolimus*.

### Cost Analysis

Here, we report standardized mean payer-relevant costs; actual average costs per patient at an individual level are reported in Supplementary Tables 5–8.

#### ND, PD, and Exacerbation Event Subgroups

ND patients incurred higher mean all-cause costs compared with PD patients ($26,419.20, ND; $24,941.47, PD) (Table 6) (median costs: $7,300.27, ND; $6,681.28, PD; *p* < 0.001). Mean direct costs for gMG treatments were similarly higher for ND patients ($9,890.37) compared with PD patients ($9,186.47). As expected, patients in the exacerbation event subgroup incurred higher all-cause costs ($43,734.15) and direct costs for gMG treatments ($21,550.02) compared with the overall ND and PD cohorts.

**Table 6:** Standardized mean 12-month payer-relevant medical and pharmacy service costs per patient in ND, PD, and exacerbation event subgroups.

**Type of costs (USD)**	**ND (*n* = 12,822)**	**PD (*n* = 29,118)**	**Exacerbation event (*n* = 4,355)**
**Mx estimated paid amount**			
All-cause costs	21,785.29	20,062.10	37,724.34
Direct costs for gMG treatments^*^	8,627.10	7,579.34	19,320.15
Unspecified gMG costs	7,676.81	7,788.92	12,123.51
**Rx paid amount**			
All-cause costs	4,633.91	4,879.36	6,009.82
Direct costs for gMG treatments^*^	1,263.27	1,607.14	2,229.87
Unspecified gMG costs	NA	NA	NA
**Total paid amount**			
All-cause costs	26,419.20	24,941.47	43,734.15
Direct costs for gMG treatments^*^	9,890.37	9,186.47	21,550.02
Unspecified gMG costs	7,676.81	7,788.92	12,123.51

*gMG, generalized myasthenia gravis; Mx, medical services; NA, not applicable; ND, newly diagnosed; PD, previously diagnosed; Rx, pharmacy; USD, United States dollar*.

* *Direct costs for gMG treatments were calculated based on therapies relevant to gMG only. These were defined as intravenous immunoglobulin (IVIg) or subcutaneous immunoglobulin (SCIg), rituximab, eculizumab, plasma exchange (PLEX), acetylcholinesterase (AChE) inhibitors, non-steroidal immunosuppressive treatments (NSISTs), or corticosteroids*.

For drug costs (Table 7), IVIg and SCIg costs represented 52.8% of total direct costs for gMG treatments for ND patients ($5,223.70 of $9,890.37) and 73.4% for PD patients ($6,743.17 of $9,186.47) despite only a small proportion of patients (7.9% of ND and 8.1% of PD) receiving IVIg or SCIg treatment (Table 4). IVIg and SCIg costs also comprised the highest proportion of drug costs for the exacerbation event subgroup despite only 17.3% of patients in the subgroup being prescribed IVIg or SCIg. Eculizumab incurred the second highest costs among drug classes for both the ND patient cohort and the exacerbation event subgroup, but not for the PD patient cohort.

**Table 7:** Standardized mean 12-month payer-relevant drug costs per patient in ND, PD, and exacerbation event subgroups.

**Type of costs (USD)**	**ND (*n* = 12,822)**	**PD (*n* = 29,118)**	**Exacerbation event (*n* = 4,355)**
**Mx estimated paid amount**			
IVIg or SCIg	4,954.15	6,299.06	12,757.53
Rituximab	312.59	514.66	894.45
Eculizumab	3,203.47	535.38	5,263.24
PLEX	150.78	220.60	396.59
AChE inhibitors	2.23	0.43	0.55
NSISTs^*^	2.45	7.57	3.39
Corticosteroids	1.44	1.63	4.42
**Rx paid amount**			
IVIg or SCIg	269.55	444.11	723.43
Rituximab	7.02	10.90	6.24
Eculizumab	133.99	83.77	361.49
PLEX	-	0.02	-
AChE inhibitors	570.00	700.03	749.95
NSISTs^*^	246.73	337.65	347.79
Corticosteroids	35.97	30.66	40.98
**Total paid amount**			
IVIg or SCIg	5,223.70	6,743.17	13,480.95
Rituximab	319.61	525.55	900.68
Eculizumab	3,337.46	619.16	5,624.73
PLEX	150.78	220.62	396.59
AChE inhibitors	572.23	700.47	750.50
NSISTs^*^	249.18	345.22	351.18
Corticosteroids	37.41	32.29	45.40

*AChE, acetylcholinesterase; IVIg, intravenous immunoglobulin; Mx, medical services; ND, newly diagnosed; NSIST, non-steroidal immunosuppressive treatment; PD, previously diagnosed; PLEX, plasma exchange; Rx, pharmacy; SCIg, subcutaneous immunoglobulin; USD, United States dollar*.

* *NSISTs included azathioprine, cyclophosphamide, cyclosporine, methotrexate, mycophenolate, and tacrolimus*.

#### Crisis Event Subgroup

For the crisis event subgroup, all-cause costs were significantly higher during the 12 months immediately preceding a crisis event ($49,236.68) compared with 24–13 months ($24,354.37) prior to the crisis event (*p* < 0.001) (Table 8). All-cause costs further increased more than 3-fold in the 12 months following the crisis index date ($173,956.99 [period included the crisis event duration]; *p* < 0.001). Direct costs for gMG treatments varied across the time periods but were highest during the 12 months following the crisis index date. Unspecified gMG costs also rose significantly during the 12 months preceding the crisis event and continued to increase in the 12 months following the crisis index date (*p* < 0.001). Notably, unspecified gMG costs comprised a higher proportion of all-cause costs at most time points for the crisis event subgroup compared with ND, PD, or exacerbation event subgroups.

**Table 8:** Standardized mean 12-month payer-relevant medical and pharmacy service costs per patient in the crisis event subgroup.

**Type of costs (USD)**	**Pre-crisis (*****n*** **=** **206)**			Crisis event^*^(*n* = 206)	**Post-crisis^**†**^ (*n* = 206)**
	**36–25 months**	**24–13 months**	**12–0 months**		**0–12 months**
**Mx estimated paid amount**					
All-cause costs	15,217.29	18,710.30	44,356.68	92,586.90	168,766.58
Direct costs for gMG treatments^#x02021;^	2,604.48	6,013.05	5,141.30	353.69	23,569.42
Unspecified gMG costs	7,686.42	7,876.30	29,470.02	86,088.68	127,362.95
**Rx paid amount**					
All-cause costs	5,617.30	5,644.07	4,880.00	91.13	5,190.41
Direct costs for gMG treatments^‡^	2,717.65	2,554.05	1,908.58	21.15	1,505.87
Unspecified gMG costs	NA	NA	NA	NA	NA
**Total paid amount**					
All-cause costs	20,834.59	24,354.37	49,236.68	92,678.02	173,956.99
Direct costs for gMG treatments^‡^	5,322.13	8,567.10	7,049.88	374.84	25,075.29
Unspecified gMG costs	7,686.42	7,876.30	29,470.02	86,088.68	127,362.95

*gMG, generalized myasthenia gravis; Mx, medical services; NA, not applicable; Rx, pharmacy; USD, United States dollar*.

* *Crisis event costs were evaluated during hospital stay. As hospital expenses were coded under diagnosis-related groups (DRGs), costs may show as bundled in claims data, limiting distinct identification of individual cost items during crisis hospitalization episodes*.

† *Post-crisis period included the crisis duration*.

‡ *Direct costs for gMG treatments were calculated based on therapies relevant to gMG only. These were defined as intravenous immunoglobulin (IVIg) or subcutaneous immunoglobulin (SCIg), rituximab, eculizumab, plasma exchange (PLEX), acetylcholinesterase (AChE) inhibitors, non-steroidal immunosuppressive treatments (NSISTs), or corticosteroids*.

Similar to the ND, PD, and exacerbation event subgroups, the majority of drug costs for patients in the crisis event subgroup were attributed to IVIg or SCIg during all time periods analyzed, with a significant and marked increase in cost observed between the 12 months preceding crisis ($4,499.59) and the post-crisis period, which included the crisis duration ($12,488.30; *p* < 0.001) (Table 9). During the post-crisis period, eculizumab costs comprised the second highest proportion of drug costs despite being prescribed to only 2.4% of patients in this subgroup (Table 5). Costs incurred for all gMG-related treatment classes examined in this study were higher in the post-crisis time period compared with 36–25 months prior to a crisis event.

**Table 9:** Standardized mean 12-month payer-relevant drug costs per patient in the crisis event subgroup.

**Type of costs (USD)**	**Pre-crisis (*****n*** **=** **206)**			Crisis event^*^(*n* = 206)	**Post-crisis^**†**^ (*n* = 206)**
	**36–25 months**	**24–13 months**	**12–0 months**		**0–12 months**
**Mx estimated paid amount**					
IVIg or SCIg	1,721.95	4,912.84	3,808.33	206.23	12,488.30
Rituximab	260.30	416.47	130.15	-	1,688.72
Eculizumab	-	-	-	-	6,948.66
PLEX	604.88	681.58	1,200.74	147.41	2,411.94
AChE inhibitors	0.07	0.04	0.23	-	0.11
NSISTs^‡^	15.27	0.13	1.04	-	23.83
Corticosteroids	2.02	1.99	0.82	0.05	7.87
**Rx paid amount**					
IVIg or SCIg	1,972.99	1,441.13	691.26	-	-
Rituximab	-	137.44	198.95	-	-
Eculizumab	-	-	-	-	-
PLEX	-	-	-	-	-
AChE inhibitors	585.33	812.45	844.40	14.05	884.19
NSISTs^‡^	137.50	139.58	152.59	5.89	570.36
Corticosteroids	21.83	23.45	21.37	1.21	51.32
**Total paid amount**					
IVIg or SCIg	3,694.94	6,353.96	4,499.59	206.23	12,488.30
Rituximab	260.30	553.91	329.10	-	1,688.72
Eculizumab	-	-	-	-	6,948.66
PLEX	604.88	681.58	1,200.74	147.41	2,411.94
AChE inhibitors	585.40	812.49	844.63	14.05	884.30
NSISTs^‡^	152.77	139.71	153.63	5.89	594.19
Corticosteroids	23.85	25.44	22.19	1.26	59.18

*AChE, acetylcholinesterase; IVIg, intravenous immunoglobulin; Mx, medical services; NSIST, non-steroidal immunosuppressive treatment; PLEX, plasma exchange; Rx, pharmacy; SCIg, subcutaneous immunoglobulin; USD, United States dollar*.

* *Crisis event costs were evaluated during hospital stay. As hospital expenses were coded under diagnosis-related groups (DRGs), costs may show as bundled in claims data, limiting distinct identification of individual cost items during crisis hospitalization episodes*.

† *Post-crisis period included the crisis duration*.

‡ *NSISTs included azathioprine, cyclophosphamide, cyclosporine, methotrexate, mycophenolate, and tacrolimus*.

## Discussion

The present analysis of HCRU and costs associated with gMG in the US was informed through a robust algorithm validated by clinical experts to capture patients living with gMG within national claims data from Symphony Health's IDV® dataset. Although gMG has been associated with a substantial clinical and economic burden, few studies have highlighted the magnitude of this burden in the US (11, 17–23) despite having the highest direct medical cost of MG out of 8 countries with available data (10). A need for additional and updated studies—particularly focusing on patients' clinical characteristics—was reported in a recent systematic literature review (10). To address these knowledge gaps, in addition to evaluating HCRU, treatment patterns, and costs in patients with gMG who were ND, PD, and who experienced exacerbation events, we expanded our analysis for patients who experienced myasthenic crisis events to include 3 years leading up to the crisis event, during the crisis event, and 1 year following the crisis event.

Though values are not directly comparable due to differences in databases used, time periods analyzed, and cost estimation methodologies, mean per-patient annual direct medical costs for our larger study population ($26,419.20 for ND; $29,941.47 for PD) were in a consistent range with a previous US-based burden of illness report for MG ($28,780) (10, 11). Other past analyses using claims data from Symphony Health's IDV® dataset may aid in further contextualizing our findings in gMG. Mean 1-year per-patient all-cause costs from IDV® in 10,140 patients with epilepsy without tuberculosis sclerosis complex was reported to be $56,397 (24), while total cost over 5 years was reported at $47,464 (average of $9492.80 per year) for 15,599 patients with rheumatoid arthritis who used oral methotrexate only (25). For 7043 patients with chronic obstructive pulmonary disease, 1-year all-cause costs from IDV® were reported at $19,690.40 (26). Though direct comparisons cannot be made due to non-trivial differences in cost estimation algorithms (i.e., charged vs. paid amounts), it is apparent that costs for gMG lie in the higher range within these relatively age-matched chronic conditions, with exacerbations and crisis events causing costs to rise even further. Of note, previous reporting that the cost of MG management comprised 78% of total direct medical costs when compared with a matched non-MG control group suggests that the majority of healthcare expenses observed in our study can be attributed to gMG management (17).

During interviews, clinical experts estimated the prevalence of gMG to be higher than that found in the literature and claimed that almost all patients require therapy at some point. They predicted that differential diagnoses, diagnostic delays, and the complexity of treatment decisions for ND gMG might lead to increased intervention and thus, increased healthcare costs when compared with PD gMG. Consistent with this, our results demonstrated higher 12-month HCRU (Table 2) and costs (Table 6) associated with ND gMG compared with PD gMG. Increased HCRU was impacted by greater mean hospitalizations, longer LOS, and greater mean ED visits, and increased costs were largely attributed to medical and pharmacy service costs. The pronounced proportion of ND patients with high HCRU associated with the time of new diagnosis suggests that previously undiagnosed patients may present with more severe symptoms or complications that require more intense clinical attention compared with PD patients.

A granular analysis of patients who experienced myasthenic crisis events yielded intriguing results. During the 36–25 months and 24–13 months preceding the crisis index date, HCRU and costs incurred were comparable to, or even lower than, corresponding results for ND and PD patient cohorts. However, a dramatic increase in HCRU and costs was observed during the year immediately preceding the crisis event, which further increased during the year following the crisis index date (which included crisis duration). Increased usage of rescue treatments including IVIg or SCIg, PLEX, eculizumab, and rituximab during these time periods contributed most to the increased costs (Table 9). These data suggest that myasthenic crisis events, which are key drivers of HCRU and cost for patients living with gMG, may be predictable and preventable; additional investigation around possible windows of opportunity and appropriate interventions are warranted.

Consistent with recommended treatment patterns in the literature (27), most patients in the study were observed to be undergoing treatment with AChE inhibitors, corticosteroids, and/or NSISTs (Table 4). Increased usage of monoclonal antibodies (eculizumab and rituximab), recommended for patients with severe symptoms with insufficient response to standard treatment (28), was observed in the exacerbation and crisis event subgroups. Across all subgroups, costs for IVIg or SCIg treatment accounted for the greatest proportion of total drug costs (Tables 7, 9), despite a small subset of patients utilizing them (Tables 4, 5). Our results are consistent with a previous study reporting that IVIg accounted for 85% of MG-related pharmacy costs despite being used by 12% of patients with MG (11). Of note, the present study did not differentiate between rescue and maintenance IVIg treatment, and associated cost differences have not been investigated. Further, our study did not address the treatment-refractory patient subgroup as other recent studies have focused on examining their burden in detail (7–9, 29, 30).

A major limitation in the present study pertains to defining exacerbation events. Using solely ICD diagnostic codes as a proxy for gMG exacerbations can involve considerable caveats, as misuse or misclassification of diagnostic codes can occur; ICD codes for MG with or without acute exacerbation may be used interchangeably by mistake, and their usage may not totally reflect MG status. This is an inherent limitation associated with analysis of any administrative health claims data without a link to extensive medical records. Additionally, the occurrence of exacerbations can be subjective depending on the individual patient's history, rate of disease progression, and bulbar involvement, and etiologies associated with exacerbation claims were not available in the data. Considering these factors, it should be noted that this subgroup may not capture those with true exacerbations to the full extent. Nevertheless, we found that this subgroup, which included a considerable proportion of patients, had higher HCRU and costs compared with the ND and PD patient cohorts. Despite the limitations, higher usage of later-line treatments (e.g., IVIg, SCIg, and PLEX) suggests this distinct subgroup of patients had a clearly more severe disease profile requiring greater clinical intervention compared with the overall population of patients with gMG.

Some other limitations in our study should be noted. First, the mean age range of the study population is higher compared with some previous studies (11, 21). Though the number of late-onset MG diagnoses are rapidly increasing (18, 27, 28), this alone cannot account for the gap. We confirmed that while our initial cohort had a mean age more aligned to a previous insurance database study (11), the mean age increased as each of the following study inclusion criteria were applied: (1) having at least 2 MG claims at least 30 days apart, (2) adults aged ≥18 years, (3) index date between 2017 and 2018, and (4) continuous quarterly claims activity. While we recognize that these criteria led to the enrichment of older patients who may seek increased medical care while excluding a proportion of younger and healthier patients, they were critical to ensure a robust analysis and to maintain consistency with standard practice for longitudinal analyses of open claims data. Next, the present study could not account for key socioeconomic and demographic factors unavailable in the dataset (including race and ethnicity) that are additional underlying drivers of resource use and costs. Critical factors pertaining to disease severity, including MG activities of daily living (MG-ADL), the Myasthenia Gravis Foundation of America (MGFA) class, and MGFA post-intervention status (PIS) are also not available in claims data and must be investigated through other databases. However, some additional patient subgroup stratifications that are feasible using available data in claims (including age, gender, key comorbidities, and geographic location) are currently being analyzed in a separate ongoing follow-up study to address further knowledge gaps pertaining to the burden of gMG in various patient subpopulations.

Further, claims are subject to inconsistencies in diagnostic and procedural coding practices; although the inclusion and exclusion criteria sought to limit cases involving misdiagnoses (e.g., chronic fatigue syndrome or fibromyalgia), they did not account for patients with gMG who had not received a diagnosis within the time periods of interest. Any change in the patients' point-of-care location or benefits enrollment during a quarter with claims activity, as well as any services provided outside of the Symphony Health (Blue Bell, PA, USA) provider network, may have led to missed events. Next, though our analysis took multiple measures to exclude patients with oMG with added considerations for disease progression from oMG to gMG, separate diagnostic codes between oMG and gMG are necessary to establish further targeted insights. Additionally, the IDV® dataset was provider-based (rather than insurance-based) with records unavailable for patient eligibility. Medical expenditures were reported as charged amounts, which are different from actual paid amounts. To address these limitations in the dataset, expert guidance was incorporated into our unique cost estimation algorithms elaborated in Supplementary Methods. Lastly, our definition for direct costs for gMG treatments did not capture costs associated with HCRU, other medical services, or the management of comorbidities. Further comparison of patients with gMG against a claims-based non-gMG population will lend insights into the incremental costs and HCRU of gMG management relative to a general population.

In the present study of patients living with gMG, we observed that ND patients had a higher HCRU and cost burden compared with PD patients, which could be attributed to severe and/or progressed disease symptoms requiring robust treatment, or confounding symptoms leading to differential diagnoses, diagnostic delays, and the complexity of treatment decisions. Additional research is required to better understand the potential drivers of increased HCRU and costs of exacerbation and crisis events by delineating socioeconomic factors and intensity of interventions. Furthermore, real-world reimbursement data should be utilized to validate our novel algorithm. To lower costs and improve outcomes for patients with gMG, providers can monitor and identify risk factors for crisis events to achieve prevention or timely intervention. Importantly, there is a need to reduce IVIg and SCIg costs which account for a major portion of cost to payers, and preventive treatment options that could minimize the risk of crisis events should be made available to relieve the cost burden of patients with gMG in the US.

## Data Availability Statement

The data analyzed in this study was obtained from Symphony Health (https://symphonyhealth.prahs.com/), the following licenses/restrictions apply: The data analyzed in this study are subject to restrictions due to a license agreement between Symphony Health (Blue Bell, PA, USA) and ZS Associates (Evanston, IL, USA). Requests to access these datasets should be directed to Symphony Health, https://symphonyhealth.prahs.com/contact/inquiry.

## Ethics Statement

Ethical review and approval was not required for the study on human participants in accordance with the local legislation and institutional requirements. Written informed consent for participation was not required for this study in accordance with the national legislation and the institutional requirements.

## Author Contributions

CA, AG, AW, and SB were involved in data analysis, statistical analysis, and drafting the manuscript. GP, YL, DG, and EB contributed to data interpretation and refining methods. All authors were involved in developing the concept and designing the study methodology, contributed to the article, and approved the submitted version.

## Funding

This study was funded by argenx US Inc., Boston, MA, USA. The funder was involved in the study design, interpretation of aggregate results, manuscript reviews, and the decision to submit for publication. The funder did not have access to raw data obtained from Symphony Health, Blue Bell, PA, USA.

## Conflict of Interest

GP, DG, and EB are employees of argenx US Inc., Boston, MA, United States. YL is a paid consultant for and receives grant support from argenx US Inc., Boston, MA, United States. CA, AG, AW, and SB are employees of ZS Associates New York, NY, United States; Princeton, NJ, United States; Bangalore, India and serve as paid consultants for argenx US Inc., Boston, MA, United States.

## Publisher's Note

All claims expressed in this article are solely those of the authors and do not necessarily represent those of their affiliated organizations, or those of the publisher, the editors and the reviewers. Any product that may be evaluated in this article, or claim that may be made by its manufacturer, is not guaranteed or endorsed by the publisher.

## Abbreviations

AChE: acetylcholinesterase
COVID-19: coronavirus disease 2019
CPI-U: Consumer Price Index for All Urban Consumers
CPT: Current Procedural Terminology
ED: emergency department
GERD: gastroesophageal reflux disease
gMG: generalized MG
HCRU: healthcare resource utilization
ICD: International Classification of Diseases
ICU: intensive care unit
IDV®: Integrated Dataverse®
IVIg: intravenous immunoglobulin
LOS: length of stay
LRP4: low-density lipoprotein receptor-related protein 4
MG: myasthenia gravis
MG-ADL: MG activities of daily living
MGFA: Myasthenia Gravis Foundation of America
MuSK: muscle-specific kinase
NDC: National Drug Code
NMJ: neuromuscular junction
NSIST: non-steroidal immunosuppressive treatment
oMG: ocular MG
PIS: post-intervention status
PLEX: plasma exchange
SCIg: subcutaneous immunoglobulin
SD: standard deviation
US: United States.
