# Supplementary material for: Real-World Healthcare Resource Utilization and Cost Burden Assessment for Adults With Generalized Myasthenia Gravis in the United States
Source: Front Neurol. 2022 Jan 18;12:809999. doi: 10.3389/fneur.2021.809999 (PMC8805609; doi:10.3389/fneur.2021.809999)
Supplement: Supplementary file 1 [file Data_Sheet_1.docx]

Supplementary Material

**Contents**

[1 Supplementary Methods 3](#_Toc89529659)

[1.1 Expert interview structure 3](#_Toc89529660)

[1.2 Medical service costs and pharmacy costs 3](#_Toc89529661)

[1.3 Cost estimation methods 3](#_Toc89529662)

[1.4 Outlier removal using sigma ranges 4](#_Toc89529663)

[2 Supplementary Figures 6](#_Toc89529664)

[Supplementary Figure 1 6](#_Toc89529665)

[3 Supplementary Tables 7](#_Toc89529666)

[Supplementary Table 1. Diagnostic codes used to identify patients with myasthenia gravis for initial screening 7](#_Toc89529667)

[Supplementary Table 2: Inclusion and exclusion criteria for patient subgroups 8](#_Toc89529668)

[Supplementary Table 3: Distribution of common comorbidities among patient subgroups 9](#_Toc89529669)

[Supplementary Table 4: Quarterly hospitalizations and LOS in ND patients over 12 months after the index dates 10](#_Toc89529670)

[Supplementary Table 5: Actual mean 12-month payer-relevant medical and pharmacy service costs per patient in ND, PD, and exacerbation event subgroups 11](#_Toc89529671)

[Supplementary Table 6: Actual mean 12-month payer-relevant drug costs per patient in ND, PD, and exacerbation event subgroups 12](#_Toc89529672)

[Supplementary Table 7: Actual mean 12-month payer-relevant medical and pharmacy service costs per patient in the crisis event subgroup 13](#_Toc89529673)

[Supplementary Table 8: Actual mean 12-month payer-relevant drug costs per patient in the crisis event subgroup 14](#_Toc89529674)

[4 Statistical Analysis Results 15](#_Toc89529675)

[Supplementary Table 2S. Standardized healthcare resource utilization in ND and PD subgroups over 12 months 15](#_Toc89529676)

[Supplementary Table 3S. Standardized healthcare resource utilization in the crisis event subgroup 16](#_Toc89529677)

[Supplementary Table 5S. Patient distribution within each drug class for the crisis event subgroup 17](#_Toc89529678)

[Supplementary Table 8S. Standardized mean 12-month payer-relevant medical and pharmacy service costs per patient in the crisis event subgroup 18](#_Toc89529679)

[Supplementary Table 9S. Standardized mean 12-month payer-relevant drug costs per patient in the crisis event subgroup 19](#_Toc89529680)

# Supplementary Methods

## Expert interview structure

One week prior to the interview, a pre-read containing the following information was sent to each expert:

- Key research objectives of the manuscript
- Published information on the epidemiology of myasthenia gravis (MG)
- Treatment pathways and options for patients with generalized MG (gMG) per published literature and international consensus guidelines
- Study design and subgroup definitions

Experts were interviewed individually during virtual 1-hour sessions conducted over video conference. Interviews focused on the experts’ clinical experience in relation to published data and were structured in the following manner:

- Disease epidemiology (10 minutes)
- Treatment patterns and guidelines in gMG (15 minutes)
- Healthcare resource utilization (HCRU) and costs in gMG (15 minutes)
- Challenges and considerations for burden of illness analyses using claims data (20 minutes)

Inputs from the interviews were used to refine the development of our research methodology and cost estimation methods.

## Medical service costs and pharmacy costs

Medical service costs were defined as cost incurred for services and procedures administered in inpatient, outpatient, clinic, and office settings.

Pharmacy costs were defined as costs incurred for prescription drugs including amounts paid by patients and their respective health plans. Patient out-of-pocket costs were determined by their respective health plans which returned total amount owed to the pharmacy (including copays, amounts applied to deductibles, and over maximum amount penalties). Plan pay was defined as the total amount to be paid by a health plan including ingredient costs, incentive fees, and dispensing fees.

## Cost estimation methods

In the IDV^®^ dataset, only the charged amount for procedures performed was available. In order to derive estimated paid amounts, an approach was designed based on expert input to utilize the Centers for Medicare & Medicaid Services (CMS) allowed amount (Physician and Other Supplier Data CY 2018, available at: https://www.cms.gov/research-statistics-data-systems/medicare-provider-utilization-and-payment-data/medicare-provider-utilization-and-payment-data-physician-and-other-supplier/physician-and-other-supplier-data-cy-2018). Payment and charged amounts for procedure codes were extracted from the Medicare National Healthcare Common Procedure Coding System (HCPCS) Aggregate Summary Table CY 2018 dataset (hereafter referred to as Medicare dataset) to derive the CMS allowed amount for the procedure codes in the IDV^®^ dataset, which acted as an approximation for the amount paid by payers for procedures. For commercial claims, CMS allowed amounts on average across all claim line items were doubled, based on approximations by experts who were interviewed.

The paid amount for procedures was calculated using the following steps. First, primary and non-primary line items in a claim based on highest charged amount were identified. Of note, when providers report more than a single procedure during a single encounter (Verhovshek J. Understanding the multiple procedure rule. Advancing the Business of Healthcare Website <https://www.aapc.com/blog/27973-understanding-the-multiple-procedure-rule/>), payers typically reimburse only the highest-valued procedure at the full fee schedule value and reduce payment for the second and subsequent procedures, as payers reason that many of the component services that comprise the physician’s work (such as surgical approach and closure) should be paid only once, per session. Thus, the line item with the highest charged amount was designated as the primary line item, and rest of the line items were designated as non-primary line items.

Second, procedure codes from the IDV^®^ dataset were mapped to those from the Medicare dataset. The Medicare dataset contained 8,830 distinct procedure codes including the HCPCS Drug Indicator, Place of Service, Average Submitted Charged Amount, Average Medicare Allowed Amount, and Average Medicare Payment Amount; these procedure codes were matched to those from the IDV^®^ dataset in order to calculate the paid amount.

Third, paid amounts were calculated for procedure codes that were mapped with the Medicare dataset. Paid amount for the primary line item was defined as (CMS allowed amount) × (number of units) × 100%, and paid amount for a non-primary item was defined as (CMS allowed amount) × (number of units) × 50%. The stated number of units were applied if the procedure code was "drug-intensive" (e.g., “J1561: Injection, immune globulin, (gamunex-c/gammaked), non-lyophilized, 500 mg”). One unit was applied if the procedure code was "service-intensive" (e.g., “99214: Office or other outpatient visit for evaluation and management”).

Next, paid amounts were calculated for procedure codes that were not mapped with the Medicare dataset. Paid amount for procedure codes that were unavailable or unmapped in the IDV^®^ dataset was defined as (total charged amount) × (ratio of total CMS allowed amount to total charged amount). Paid amount and ratios were calculated based on the place of service and type of therapy.

Final paid amounts considering all records were defined as the total sum of paid amounts for primary and non-primary claims as well as claims from unavailable and unmapped procedure codes. Based on expert feedback, payer-relevant costs were adjusted and defined as 2 × (paid amount) for patients on commercial plans.

## Outlier removal using sigma ranges

Claims data were segregated as drug-intensive or service-intensive using the HCPCS Drug Indicator field in the CMS pricing file. For service-intensive procedure claims, a value of 1 unit administered was imputed, irrespective of the value in the data. For drug-intensive claims, standard deviations (σ) and mean units administered for each cohort were calculated.

Any patients with claim(s) having units administered beyond 6 standard deviations from the average units administered in the cohort were removed from the analysis. This resulted in a drop-off of 17 newly diagnosed patients and 35 previously diagnosed patients from the study population, one of whom fulfilled inclusion criteria for the crisis event subgroup.

# Supplementary Figures

## Supplementary Figure 1

**Initial cohort: n=168,600**

**ND: n=23,109**

Patients with first diagnostic claim for MG filed between January 1, 2017 and December 31, 2018​

**Final ND gMG cohort**

**n=12,822**

**Final PD gMG cohort**

**n=29,118**

**PD: n=41,450**

Patients with first diagnostic claim for MG filed before January 1, 2017

AND an MG diagnostic claim filed from January 1, 2017 to December 31, 2018​

**Study sample patient cohort: n=66,119**

Patients with MG diagnostic claim filed between January 1, 2017 and December 31, 2018

**Crisis event subgroup**

**n=206**

(44.2% ND; 55.8% PD)

+ Continuous quarterly claims activity of −1/+3 years

**Exacerbation event subgroup**

**n=4,355**

(51.4% ND; 48.6% PD)

+ Continuous quarterly claims activity of −1/+3 years

Neither

n=37,379

**Total: n=41,940**

Patients with continuous quarterly claims activity of +1 year

**Supplementary Figure 1.** Cohort diagram for the final study population and subgroups. From the 168,600 patients initially identified with MG diagnoses in the dataset, 66,119 had claims filed between January 1, 2017 and December 31, 2018. A subset of 1560 patients were excluded from this cohort as their claims were associated with only ophthalmologic specialists. From the remaining population, patients were identified as “newly diagnosed” (ND) with gMG during the study period, or as first diagnosed with gMG prior to the study period (“previously diagnosed or PD”). Patients with continuous quarterly claims activity over 12 months (12,822 and 29,1118 patients, respectively) were included in the final analyses (total, n=41,940). Patients who met criteria for the exacerbation event (4,355 patients) or crisis event (206 patients) subgroups were drawn from the final study population regardless of when they were first diagnosed with gMG. gMG, generalized MG; MG, myasthenia gravis; ND, newly diagnosed; PD, previously diagnosed.

# Supplementary Tables

## Supplementary Table 1. Diagnostic codes used to identify patients with myasthenia gravis for initial screening

| **ICD code type** | **Diagnosis code** | **Diagnosis description** |
| --- | --- | --- |
| ICD-10 | G70.00 | Myasthenia gravis without (acute) exacerbation |
|  | G70.01 | Myasthenia gravis with (acute) exacerbation |
|  | G70.02^*^ | Congenital and developmental myasthenia |
| ICD-9 | 358.00 | Myasthenia gravis without (acute) exacerbation |
|  | 358.01 | Myasthenia gravis with (acute) exacerbation |

ICD-9, International Classification of Diseases, Ninth Revision; ICD-10, International Classification of Diseases, Tenth Revision.

^*^Patients with ICD code G70.02 did not have a major impact on results as patients less than 18 years of age did not meet the study inclusion criteria.

## Supplementary Table 2: Inclusion and exclusion criteria for patient subgroups

| **Subgroup** | **Inclusion criteria** | **Exclusion criteria** |
| --- | --- | --- |
| ND | - First diagnostic claim for gMG filed between January 2017 and December 2018 | - Patients with no continuous quarterly activity 12 months prior to and after index date |
| PD | - First diagnostic claim for gMG filed before January 2017, AND - A diagnostic claim for gMG filed from January 2017 to December 2018 |  |
| Exacerbation event | - Patients with a gMG diagnostic claim, AND an acute exacerbation claim (ICD codes G70.01 or 358.01) filed between January 2017 and December 2018 | - Patients identified in crisis event cohort - Patients having intubation claims - Patients with no continuous quarterly activity 36 months prior to and 12 months following the exacerbation index date |
| Crisis  event | - Patients with a gMG diagnostic claim, AND - ≥1 claim for intubation filed from January 2017 to December 2018, AND - An inpatient stay either on the same date the claim was filed, or the day after, AND - ≥1 claim corresponding to ICU admission on the same, previous, or next day of receiving intubation, identified using the following CPT codes: - 99291- CRITICAL CARE ILL/INJURED PATIENT INIT 30-74 MIN - 99292- CRITICAL CARE ILL/INJURED PATIENT ADDL 30 MIN | - Patients having first intubation claim filed before January 2017 - Patients having <2 days of hospital stay - Patients with no continuous quarterly activity 36 months prior to and 12 months following the crisis event index date |

ADDL, additional; CPT, Current Procedural Terminology; gMG, generalized myasthenia gravis; ICD, International Classification of Diseases; ICU, intensive care unit; INIT, initial; MIN, minutes; ND, newly diagnosed; PD, previously diagnosed.

## Supplementary Table 3: Distribution of common comorbidities among patient subgroups

|  | **Overall**  **(n=41,940)** | | | **ND**  **(n=12,822)** | | | **PD**  **(n=29,118)** | | |
| --- | --- | --- | --- | --- | --- | --- | --- | --- | --- |
| **Comorbidity**  **(ICD code)** | **C** | **E** | **N** | **C** | **E** | **N** | **C** | **E** | **N** |
| Essential (primary) hypertension (I10), % | 0.8 | 11.3 | 87.9 | 1.2 | 18.5 | 80.3 | 0.6 | 8.0 | 91.5 |
| Hyperlipidemia, unspecified (E78.5), % | 0.7 | 11.4 | 87.9 | 1.1 | 18.0 | 80.9 | 0.6 | 8.3 | 91.2 |
| Type 2 diabetes mellitus without complications (E11.9), % | 0.9 | 12.4 | 86.7 | 1.3 | 20.8 | 77.9 | 0.8 | 8.6 | 90.6 |
| Gastroesophageal reflux disease without esophagitis (K21.9), % | 0.9 | 11.8 | 87.3 | 1.4 | 18.5 | 80.1 | 0.7 | 8.6 | 90.7 |
| Hypothyroidism, unspecified (E03.9), % | 0.5 | 11.9 | 87.5 | 0.8 | 19.3 | 80.0 | 0.4 | 8.5 | 91.1 |

C, crisis event subgroup; E, exacerbation event subgroup; ICD, International Classification of Diseases; N, neither subgroup; ND, newly diagnosed; PD, previously diagnosed.

## Supplementary Table 4: Quarterly hospitalizations and LOS in ND patients over 12 months after the index dates

|  | **Q1**^*^ | **Q2**^*^ | **Q3**^*^ | **Q4**^*^ |
| --- | --- | --- | --- | --- |
| **Patients, n (%)** | 1,906 (62.9) | 783 (25.8) | 728 (24.0) | 683 (22.5) |
| **Hospitalizations** |  |  |  |  |
| Standardized mean | 0.22 | 0.08 | 0.08 | 0.07 |
| **LOS** |  |  |  |  |
| Standardized mean | 0.90 | 0.37 | 0.31 | 0.29 |

LOS, length of stay; ND, newly diagnosed; Q, quarter.

^*^Q1, Q2, and Q3 each contain 91 consecutive days (13 weeks each). Q4 values contain data from the last 92 consecutive days of year 1 after the index dates.

## Supplementary Table 5: Actual mean 12-month payer-relevant medical and pharmacy service costs per patient in ND, PD, and exacerbation event subgroups

| **Type of costs** | **ND**  **(n=12,822)** | **PD**  **(n=29,118)** | **Exacerbation event**  **(n=4,355)** |
| --- | --- | --- | --- |
| **Mx estimated paid amount, USD (n)** | | | |
| All-cause costs | 21,785.29 (12,822) | 20,062.10 (29,118) | 37,724.34 (4,355) |
| Direct costs for gMG treatments^*^ | 49,670.71 (2,227) | 44,050.93 (5,010) | 66,355.89 (1,268) |
| Unspecified gMG costs | 19,506.94 (5,046) | 22,259.09 (10,189) | 26,280.67 (2,009) |
| **Rx paid amount, USD (n)** | | | |
| All-cause costs | 5,505.04 (10,793) | 5,907.09 (24,052) | 7,096.73 (3,688) |
| Direct costs for gMG treatments^*^ | 1,930.35 (8,391) | 2,488.12 (18,808) | 3,173.55 (3,060) |
| Unspecified gMG costs | NA | NA | NA |
| **Total paid amount, USD (n)** | | | |
| All-cause costs | 26,419.20 (12,822) | 24,941.47 (29,118) | 43,734.15 (4,355) |
| Direct costs for gMG treatments^*^ | 14,057.68 (9,021) | 13,090.52 (20,434) | 27,530.17 (3,409) |
| Unspecified gMG costs | 19,506.94 (5,046) | 22,259.09 (10,189) | 26,280.67 (2,009) |

gMG, generalized myasthenia gravis; Mx, medical services; NA, not available; ND, newly diagnosed; PD, previously diagnosed; Rx, pharmacy; USD, United States dollar.

^*^Direct costs for gMG treatments were calculated based on therapies relevant to gMG only. These were defined as intravenous immunoglobulin (IVIg) or subcutaneous immunoglobulin (SCIg), rituximab, eculizumab, plasma exchange (PLEX), acetylcholinesterase (AChE) inhibitors, nonsteroidal immunosuppressive treatments (NSISTs), or corticosteroids.

## Supplementary Table 6: Actual mean 12-month payer-relevant drug costs per patient in ND, PD, and exacerbation event subgroups

| **Type of cost** | **ND**  **(n=12,822)** | **PD**  **(n=29,118)** | **Exacerbation event**  **(n=4,355)** |
| --- | --- | --- | --- |
| **Mx estimated paid amount, USD (n)** | | | |
| IVIg or SCIg | 67,865.49 (936) | 84,329.21 (2,175) | 78,919.07 (704) |
| Rituximab | 40,898.17 (98) | 46,395.52 (323) | 59,927.87 (65) |
| Eculizumab | 507,096.98 (81) | 318,149.64 (49) | 509,364.42 (45) |
| PLEX | 5,461.23 (354) | 9,255.71 (694) | 6,038.92 (286) |
| AChE inhibitors | 177.58 (161) | 46.27 (273) | 41.18 (58) |
| NSISTs^*^ | 218.13 (144) | 487.69 (452) | 144.62 (102) |
| Corticosteroids | 19.71 (934) | 22.87 (2,077) | 50.01 (385) |
| **Rx paid amount, USD (n)** | | | |
| IVIg or SCIg | 37,163.03 (93) | 51,933.65 (249) | 53,398.68 (59) |
| Rituximab | 18,011.43 (5) | 11,751.13 (27) | 13,579.44 (2) |
| Eculizumab | 171,802.89 (10) | 271,029.66 (9) | 174,922.75 (9) |
| PLEX | 0 | 672.56 (1) | 0 |
| AChE inhibitors | 1,127.33 (6,483) | 1,536.40 (13,267) | 1,380.98 (2,365) |
| NSISTs^*^ | 1,053.12 (3,004) | 1,223.15 (8,038) | 1,051.81 (1,440) |
| Corticosteroids | 83.11 (5,550) | 79.15 (11,277) | 81.67 (2,185) |
| **Total paid amount, USD (n)** | | | |
| IVIg or SCIg | 65,923.49 (1,016) | 82,257.02 (2,387) | 77,760.99 (755) |
| Rituximab | 41,394.72 (99) | 43,974.24 (348) | 59,431.37 (66) |
| Eculizumab | 480,818.93 (89) | 310,837.92 (58) | 462,183.09 (53) |
| PLEX | 5,461.23 (354) | 9,243.36 (695) | 6,038.92 (286) |
| AChE inhibitors | 1,120.85 (6,546) | 1,522.78 (13,394) | 1,366.96 (2,391) |
| NSISTs^*^ | 1,046.51 (3,053) | 1,222.59 (8,222) | 1,035.46 (1,477) |
| Corticosteroids | 80.13 (5,986) | 76.48 (12,293) | 84.34 (2,344) |

AChE, acetylcholinesterase; IVIg, intravenous immunoglobulin; Mx, medical services; ND, newly diagnosed; NSIST, nonsteroidal immunosuppressive treatment; PD, previously diagnosed; PLEX, plasma exchange; Rx, pharmacy; SCIg, subcutaneous immunoglobulin; USD, United States dollar.

^*^NSISTs included azathioprine, cyclophosphamide, cyclosporine, methotrexate, mycophenolate, and tacrolimus.

## Supplementary Table 7: Actual mean 12-month payer-relevant medical and pharmacy service costs per patient in the crisis event subgroup

| **Type of costs** | **Pre-crisis**  **(n=206)** | | | **Crisis event^*^**  **(n=206)** | **Post-crisis^†^**  **(n=206)** |
| --- | --- | --- | --- | --- | --- |
|  | **36 to 25 months** | **24 to 13 months** | **12 to 0**  **months** |  | **0 to 12**  **months** |
| **Mx estimated paid amount, USD (n)** | | | | | |
| All-cause costs | 16,674.26 (188) | 20,393.23 (189) | 45,687.38 (200) | 92,586.90 (206) | 168,766.58(206) |
| Direct costs for gMG treatments^‡^ | 19,871.24 (27) | 36,431.98 (34) | 21,614.44 (49) | 1,777.06 (41) | 53,345.95 (91) |
| Unspecified gMG costs | 25,538.73 (62) | 24,216.68 (67) | 56,211.33 (108) | 126,673.33(140) | 161,955.35(162) |
| **Rx paid amount, USD (n)** | | | | | |
| All-cause costs | 6,574.79 (176) | 6,720.68 (173) | 5,647.64 (178) | 302.77 (62) | 6,109.85 (175) |
| Direct costs for gMG treatments^‡^ | 5,654.90 (99) | 5,010.80 (105) | 2,788.42 (141) | 114.67 (38) | 2,231.72 (139) |
| Unspecified gMG costs | NA | NA | NA | NA | NA |
| **Total paid amount, USD (n)** | | | | | |
| All-cause costs | 21,038.85 (204) | 24,593.14 (204) | 49,476.86 (205) | 92,678.02 (206) | 173,956.99(206) |
| Direct costs for gMG treatments^‡^ | 9,877.11 (111) | 15,213.98 (116) | 9,430.36 (154) | 1,135.55 (68) | 32,083.91 (161) |
| Unspecified gMG costs | 25,538.73 (62) | 24,216.68 (67) | 56,211.33 (108) | 126,673.33(140) | 161,955.35(162) |

gMG, generalized myasthenia gravis; Mx, medical services; NA, not available; Rx, pharmacy; USD, United States dollar.

^*^Crisis event costs were evaluated during hospital stay. As hospital expenses were coded under diagnosis-related groups (DRGs), costs may show as bundled in claims data, limiting distinct identification of individual cost items during crisis hospitalization episodes. ^†^Post-crisis period included the crisis duration. ^‡^Direct costs for gMG treatments were calculated based on therapies relevant to gMG only. These were defined as intravenous immunoglobulin (IVIg) or subcutaneous immunoglobulin (SCIg), rituximab, eculizumab, plasma exchange (PLEX), acetylcholinesterase (AChE) inhibitors, nonsteroidal immunosuppressive treatments (NSISTs), or corticosteroids.

## Supplementary Table 8: Actual mean 12-month payer-relevant drug costs per patient in the crisis event subgroup

| **Type of cost** | **Pre-crisis**  **(n=206)** | | | **Crisis event^*^ (n=206)** | **Post-crisis^†^ (n=206)** |
| --- | --- | --- | --- | --- | --- |
|  | **25 to 36 months** | **13 to 24 months** | **0 to 12**  **months** |  | **0 to 12**  **months** |
| **Mx estimated paid amount, USD (n)** | | | | | |
| IVIg or SCIg | 59,120.20 (6) | 92,004.02 (11) | 52,301.01 (15) | 21,241.20 (2) | 85,752.99 (30) |
| Rituximab | 26,810.40 (2) | 28,597.76 (3) | 26,810.40 (1) | 0 | 43,484.62 (8) |
| Eculizumab | 0 | 0 | 0 | 0 | 286,284.60 (5) |
| PLEX | 13,844.96 (9) | 28,081.17 (5) | 19,027.07 (13) | 820.74 (37) | 9,374.69 (53) |
| AChE inhibitors | 14.61 (1) | 8.86 (1) | 15.93 (3) | 0 | 7.52 (3) |
| NSISTs^‡^ | 1,572.87 (2) | 12.96 (2) | 53.45 (4) | 0 (0) | 613.60 (8) |
| Corticosteroids | 29.76 (14) | 18.60 (22) | 5.81 (29) | 4.90 (2) | 73.65 (22) |
| **Rx paid amount, USD (n)** | | | | | |
| IVIg or SCIg | 101,609.06 (4) | 59,374.48 (5) | 47,466.85 (3) | 0 | 0 |
| Rituximab | 0 | 28,312.68 (1) | 40,984.10 (1) | 0 | 0 |
| Eculizumab | 0 | 0 | 0 | 0 | 0 |
| PLEX | 0 | 0 | 0 | 0 | 0 |
| AChE inhibitors | 1,799.67 (67) | 2,292.65 (73) | 1,567.08 (111) | 152.31 (19) | 1,556.78 (117) |
| NSISTs^‡^ | 690.83 (41) | 737.29 (39) | 604.5 (52) | 202.33 (6) | 1,468.68 (80) |
| Corticosteroids | 65.17 (69) | 61.94 (78) | 45.86 (96) | 8.61 (29) | 94.39 (112) |
| **Total paid amount, USD (n)** | | | | | |
| IVIg or SCIg | 84,573.05 (9) | 81,807.29 (16) | 51,495.31 (18) | 21,241.20 (2) | 85,752.99 (30) |
| Rituximab | 26,810.40 (2) | 28,526.49 (4) | 33,897.25 (2) | 0 | 43,484.62 (8) |
| Eculizumab | 0 | 0 | 0 | 0 | 286,284.60 (5) |
| PLEX | 13,844.96 (9) | 28,081.17 (5) | 19,027.07 (13) | 820.73 (37) | 9,374.69 (53) |
| AChE inhibitors | 1,799.89 (67) | 2,292.77 (73) | 1,567.51 (111) | 152.31 (19) | 1,556.97 (117) |
| NSISTs^‡^ | 731.86 (43) | 737.95 (39) | 597.13 (53) | 202.33 (6) | 1,492.72 (82) |
| Corticosteroids | 63.81 (77) | 59.55 (88) | 41.56 (110) | 8.65 (30) | 101.60 (120) |

AChE, acetylcholinesterase; IVIg, intravenous immunoglobulin; Mx, medical services; NSIST, nonsteroidal immunosuppressive treatment; PLEX, plasma exchange; Rx, pharmacy; SCIg, subcutaneous immunoglobulin; USD, United States dollar.

^*^Crisis event costs were evaluated during hospital stay. As hospital expenses were coded under diagnosis-related groups (DRGs), costs may show as bundled in claims data, limiting distinct identification of individual cost items during crisis hospitalization episodes. ^†^Post-crisis period included crisis duration. ^‡^NSISTs included azathioprine, cyclophosphamide, cyclosporine, methotrexate, mycophenolate, and tacrolimus.

# Statistical Analysis Results

## Supplementary Table 2S. Standardized healthcare resource utilization in ND and PD subgroups over 12 months

| **Healthcare resource unit** | **ND**  **(n=12,822)** | **PD**  **(n=29,118)** | ***p*-value^†^** |
| --- | --- | --- | --- |
| **Hospitalizations** | | | |
| Patients with ≥1 filed claim, n (%) | 3,032 (23.64) | 5,472 (18.79) | **<0.001** |
| 12-month hospitalizations, standardized mean | 0.46 | 0.34 | **<0.001** |
| LOS (days), standardized mean | 1.39 | 0.99 | **<0.001** |
| **ED visits** | | | |
| Patients with ≥1 filed claim, n (%) | 4,023 (31.37) | 8,415 (28.90) | **<0.001** |
| 12-month ED visits, standardized mean | 1 | 0.88 | **<0.001** |
| **Outpatient visits** | | | |
| Patients with ≥1 filed claim, n (%) | 9,465 (73.81) | 20,707 (71.11) | **<0.001** |
| 12-month outpatient visits, standardized mean | 7.33 | 6.92 | **<0.001** |
| **Office visits** | | | |
| Patients with ≥1 filed claim, n (%) | 10,751 (83.84) | 24,110 (82.80) | **0.008** |
| 12-month office visits, standardized mean | 8.76 | 7.98 | **<0.001** |
| **Other visits^*^** | | | |
| Patients with ≥1 filed claim, n (%) | 6,224 (48.54) | 14,114 (48.47) | 0.895 |
| 12-month office visits, standardized mean | 4.25 | 4.57 | 0.756 |

ED, emergency department; LOS, length of stay; ND, newly diagnosed; PD, previously diagnosed.

^*^Other visits included care provided in settings that did not fall within other defined categories such as independent laboratories, home health agencies, hospices, etc. ^†^Chi-squared tests and Wilcoxon rank sum tests were applied.

## Supplementary Table 3S. Standardized healthcare resource utilization in the crisis event subgroup

| **Healthcare resource unit** | **Pre-crisis**  **(n=206)** | | | | | | **Post-crisis^†^**  **(n=206)** | | |  | | | | | | | | |  |
| --- | --- | --- | --- | --- | --- | --- | --- | --- | --- | --- | --- | --- | --- | --- | --- | --- | --- | --- | --- |
|  | **36 to 25 months** | **0 to 12 months** | | **12 to 0 months** | | | **0 to 12 months** | | | ***p*-value 1**^¶^ | | | ***p*-value 2**^¶^ | | | ***p*-value 3**^¶^ | | |  |
| ***p*-value legend** | **A** | **B** | | | **C** | | | **D** | | | **A vs. B** | | | **B vs. C** | | | **C vs. D** | | |
| **Hospitalizations** |  |  | | |  | | |  | | |  | | |  | | |  | | |
| Patients with ≥1 filed claim, n (%) | 47  (22.81) | 52  (25.24) | | | 125  (60.68) | | | 206  (100) | | | 0.500 | | | **<0.001** | | | **<0.001** | | |
| 12-month hospitalizations, standardized mean | 0.46 | 0.50 | | | 1.35 | | | 2.78 | | | 0.523 | | | **<0.001** | | | **<0.001** | | |
| LOS (days), standardized mean | 1.27 | 1.47 | | | 3.60 | | | 10.14 | | | 0.543 | | | **<0.001** | | | **<0.001** | | |
| **ED visits** |  | |  | | |  | | |  | | |  | | |  | | |  | |
| Patients with ≥1 filed claim, n (%) | 62  (30.09) | | 74  (35.92) | | | 133  (64.56) | | | 206  (100) | | | 0.115 | | | **<0.001** | | | **<0.001** | |
| 12-month ED visits, standardized mean | 1.24 | | 1.34 | | | 3.01 | | | 8.78 | | | 0.216 | | | **<0.001** | | | **<0.001** | |
| **Outpatient visits** |  | |  | | |  | | |  | | |  | | |  | | |  | |
| Patients with ≥1 filed claim, n (%) | 141  (68.44) | | 142  (68.93) | | | 169  (82.03) | | | 184  (89.32) | | | 0.884 | | | **<0.001** | | | **0.007** | |
| 12-month outpatient visits, standardized mean | 7.00 | | 7.43 | | | 11.34 | | | 16.32 | | | 0.344 | | | **<0.001** | | | **<0.001** | |
| **Office visits** |  | |  | | |  | | |  | | |  | | |  | | |  | |
| Patients with ≥1 filed claim, n (%) | 156  (75.72) | | 150  (72.81) | | | 171  (83.00) | | | 169  (82.03) | | | 0.355 | | | **0.002** | | | 0.739 | |
| 12-month office visits, standardized mean | 6.31 | | 6.40 | | | 9.31 | | | 10.00 | | | 0.959 | | | **<0.001** | | | 0.137 | |
| **Other visits^‡^** |  | |  | | |  | | |  | | |  | | |  | | |  | |
| Patients with ≥1 filed claim, n (%) | 101  (49.02) | | 101  (49.02) | | | 133  (64.56) | | | 172  (83.49) | | | 1.000 | | | **<0.001** | | | **<0.001** | |
| 12-month office visits, standardized mean | 4.02 | | 4.60 | | | 6.47 | | | 15.33 | | | **0.049** | | | **0.004** | | | **<0.001** | |

ED, emergency department; LOS, length of stay.

^*^Crisis event HCRU was evaluated during hospital stay. ^†^Post-crisis period included the crisis duration. ^‡^Other visits included care provided in settings that did not fall within other defined categories such as independent laboratories, home health agencies, hospices, etc. ^§^Since visits were calculated based on respective claims, outpatient/office claims present in the data appear proportionately lower during crisis duration. ^¶^McNemar tests and Wilcoxon signed-rank tests were applied.

## Supplementary Table 5S. Patient distribution within each drug class for the crisis event subgroup

| **Therapeutic class, n (%)​** | **Pre-crisis**  **(n=206)** | | | **Post-crisis (n=206)** |  |  |  |
| --- | --- | --- | --- | --- | --- | --- | --- |
|  | **36 to 25 months** | **24 to 13 months** | **12 to 0**  **months** | **0 to 12**  **months** | ***p*-value 1^‡^** | ***p*-value 2^‡^** | ***p*-value 3^‡^** |
| ***p*-value legend** | **A** | **B** | **C** | **D** | **A vs. B** | **B vs. C** | **C vs. D** |
| IVIg + SCIg | 9 (4.4) | 16 (7.8) | 18 (8.7) | 30 (14.5) | **0.020** | 0.593 | **0.028** |
| Rituximab | 2 (1.0) | 4 (1.9) | 2 (1.0) | 8 (3.9) | 0.317 | 0.157 | **0.034** |
| Eculizumab | 0 (0) | 0 (0) | 0 (0) | 5 (2.4) | 1.000 | 1.000 | **<0.001** |
| PLEX | 9 (4.4) | 5 (2.4) | 13 (6.3) | 53 (25.7) | 0.206 | **0.005** | **<0.001** |
| AChE inhibitors | 67 (32.5) | 73 (35.4) | 111 (53.8) | 117 (56.7) | 0.273 | **<0.001** | 0.396 |
| NSISTs^†^ | 43 (20.9) | 39 (18.9) | 53 (25.7) | 82 (39.8) | 0.371 | **0.003** | **<0.001** |
| Corticosteroids | 77 (37.4) | 88 (42.7) | 110 (53.4) | 120 (58.3) | 0.138 | **0.005** | 0.211 |

AChE, acetylcholinesterase; IVIg, intravenous immunoglobulin; NSIST, nonsteroidal immunosuppressive treatment; PLEX, plasma exchange; SCIg, subcutaneous immunoglobulin.

^*^Most costs and drug utilization information during a crisis event were captured from inpatient claims. These inpatient costs are not directly attributable to gMG-specific therapies; hence, overall utilization numbers are underreported (nearly to 45%). ^†^NSISTs included azathioprine, cyclophosphamide, cyclosporine, methotrexate, mycophenolate, and tacrolimus. ^‡^McNemar tests were applied.

## Supplementary Table 8S. Standardized mean 12-month payer-relevant medical and pharmacy service costs per patient in the crisis event subgroup

| **Type of costs (USD)** | **Pre-crisis**  **(n=206)** | | | | | **Post-crisis^†^**  **(n=206)** | |  |  |  |  |
| --- | --- | --- | --- | --- | --- | --- | --- | --- | --- | --- | --- |
|  | **36 to 25 months** | | **24 to 13 months** | **12 to 0 months** | | **0 to 12 months** | | ***p*-value 1^§^** | ***p*-value 2^§^** | ***p*-value 3^§^** |  |
| ***p*-value legend** | **A** | | **B** | **C** | | **D** | | **A vs. B** | **B vs. C** | **C vs. D** |  |
| **Mx estimated paid amount** | | | | | | | | | | |  |
| All-cause costs | 15,217.29 | 18,710.30 | | | 44,356.68 | | 168,766.58 | 0.302 | **<0.001** | **<0.001** |  |
| Direct costs for gMG treatments^‡^ | 2,604.48 | 6,013.05 | | | 5,141.30 | | 23,569.42 | 0.328 | 0.428 | **<0.001** |  |
| Unspecified gMG costs | 7,686.42 | 7,876.30 | | | 29,470.02 | | 127,362.95 | 0.685 | **<0.001** | **<0.001** |  |
| **Rx paid amount** | | | | | | | | | | |  |
| All-cause costs | 5,617.30 | 5,644.07 | | | 4,880.00 | | 5,190.41 | 0.232 | 0.718 | **<0.001** |  |
| Direct costs for gMG treatments^‡^ | 2,717.65 | 2,554.05 | | | 1,908.58 | | 1,505.87 | **0.015** | 0.115 | **0.003** |  |
| Unspecified gMG costs | NA | NA | | | NA | | NA | NA | NA | NA |  |
| **Total paid amount** | | | | | | | | | | |  |
| All-cause costs | 20,834.59 | 24,354.37 | | | 49,236.68 | | 173,956.99 | 0.248 | **<0.001** | **<0.001** |  |
| Direct costs for gMG treatments^‡^ | 5,322.13 | 8,567.10 | | | 7,049.88 | | 25,075.29 | **0.030** | **0.020** | **<0.001** |  |
| Unspecified gMG costs | 7,686.42 | 7,876.30 | | | 29,470.02 | | 127,362.95 | 0.685 | **<0.001** | **<0.001** |  |

gMG, generalized myasthenia gravis; Mx, medical services; NA, not applicable; Rx, pharmacy; USD, United States dollar.

^*^Crisis event costs were evaluated during hospital stay. As hospital expenses were coded under diagnosis-related groups (DRGs), costs may show as bundled in claims data, limiting distinct identification of individual cost items during crisis hospitalization episodes. ^†^Post-crisis period included the crisis duration. ^‡^Direct costs for gMG treatments were calculated based on therapies relevant to gMG only. These were defined as intravenous immunoglobulin (IVIg) or subcutaneous immunoglobulin (SCIg), rituximab, eculizumab, plasma exchange (PLEX), acetylcholinesterase (AChE) inhibitors, nonsteroidal immunosuppressive treatments (NSISTs), or corticosteroids. ^§^Wilcoxon signed-rank tests were applied.

## Supplementary Table 9S. Standardized mean 12-month payer-relevant drug costs per patient in the crisis event subgroup

| **Type of costs (USD)** | **Pre-crisis**  **(n=206)** | | | **Post-crisis^†^**  **(n=206)** |  |  |  |  |
| --- | --- | --- | --- | --- | --- | --- | --- | --- |
|  | **36 to 25**  **months** | **24 to 13**  **months** | **12 to 0**  **months** | **0 to 12 months** | ***p*-value 1^§^** | ***p*-value 2^§^** | ***p*-value 3^§^** |  |
| ***p*-value legend** | **A** | **B** | **C** | **D** | **A vs. B** | **B vs. C** | **C vs. D** |  |
| **Mx estimated paid amount** | | | | | | | | |
| IVIg or SCIg | 1,721.95 | 4,912.84 | 3,808.33 | 12,488.30 | 0.078 | 0.862 | **<0.001** |  |
| Rituximab | 260.30 | 416.47 | 130.15 | 1,688.72 | 0.789 | 0.371 | **0.024** |  |
| Eculizumab | - | - | - | 6,948.66 | NA | NA | NA |  |
| PLEX | 604.88 | 681.58 | 1,200.74 | 2,411.94 | 0.359 | **0.019** | **<0.001** |  |
| AChE inhibitors | 0.07 | 0.04 | 0.23 | 0.11 | 1.000 | 0.584 | 1.000 |  |
| NSISTs^‡^ | 15.27 | 0.13 | 1.04 | 23.83 | 0.789 | 0.100 | 0.563 |  |
| Corticosteroids | 2.02 | 1.99 | 0.82 | 7.87 | 0.569 | 0.255 | 0.819 |  |
| **Rx paid amount** | | | | | | | | |
| IVIg or SCIg | 1,972.99 | 1,441.13 | 691.26 | - | 0.590 | 0.419 | NA |  |
| Rituximab | - | 137.44 | 198.95 | - | NA | 1.000 | NA |  |
| Eculizumab | - | - | - | - | NA | NA | NA |  |
| PLEX | - | - | - | - | NA | NA | NA |  |
| AChE inhibitors | 585.33 | 812.45 | 844.40 | 884.19 | **0.005** | 0.266 | 0.106 |  |
| NSISTs^‡^ | 137.50 | 139.58 | 152.59 | 570.36 | 0.325 | 0.506 | **<0.001** |  |
| Corticosteroids | 21.83 | 23.45 | 21.37 | 51.32 | 0.517 | 0.454 | **<0.001** |  |
| **Total paid amount** | | | | | | | | |
| IVIg or SCIg | 3,694.94 | 6,353.96 | 4,499.59 | 12,488.30 | 0.205 | 0.466 | **<0.001** |  |
| Rituximab | 260.30 | 553.91 | 329.10 | 1,688.72 | 0.361 | 0.789 | **0.024** |  |
| Eculizumab | - | - | - | 6,948.66 | NA | NA | NA |  |
| PLEX | 604.88 | 681.58 | 1,200.74 | 2,411.94 | 0.359 | **0.019** | **<0.001** |  |
| AChE inhibitors | 585.40 | 812.49 | 844.63 | 884.30 | **0.005** | 0.260 | 0.111 |  |
| NSISTs^‡^ | 152.77 | 139.71 | 153.63 | 594.19 | 0.228 | 0.441 | **<0.001** |  |
| Corticosteroids | 23.85 | 25.44 | 22.19 | 59.18 | 0.417 | 0.278 | **<0.001** |  |

AChE, acetylcholinesterase; IVIg, intravenous immunoglobulin; Mx, medical services; NSIST, nonsteroidal immunosuppressive treatment; PLEX, plasma exchange; Rx, pharmacy; SCIg, subcutaneous immunoglobulin; USD, United States dollar.

^*^Crisis event costs were evaluated during hospital stay. As hospital expenses were coded under diagnosis-related groups (DRGs), costs may show as bundled in claims data, limiting distinct identification of individual cost items during crisis hospitalization episodes. ^†^Post-crisis period included the crisis duration. ^‡^NSISTs included azathioprine, cyclophosphamide, cyclosporine, methotrexate, mycophenolate, and tacrolimus. ^§^Wilcoxon signed-rank tests were applied.
